# Preliminary clinical application of multimodal imaging combined with frameless robotic stereotactic biopsy in the diagnosis of primary central nervous system lymphoma

**DOI:** 10.1016/j.heliyon.2022.e12162

**Published:** 2022-12-09

**Authors:** Yong Tang, Yan Shi, Ling Wang, Zheng-ting Qian, You-wu Fan, He-Ming Wu, Xiang Li

**Affiliations:** a Department of Neurosurgery, Nanjing First Hospital, Nanjing Medical University, Nanjing 210006, China; b Department of Planning and Information, Jiangsu Commission of Health, Nanjing 210008, China

**Keywords:** Primary central nervous system lymphoma, Stereotaxic, Surgical robotics, Needle biopsy, Multimodal imaging

## Abstract

**Objective:**

To evaluate the clinical application of multimodal imaging combined with frameless robotic stereotactic biopsy in the diagnosis of primary central nervous system lymphoma (PCNSL).

**Methods:**

We retrospectively reviewed the clinical data of 8 patients who were considered suspected cases of PCNSL by multimodal imaging techniques. The final pathologic diagnosis were determined by the frameless robotic stereotactic biopsy. The postoperative related complications and pathological results were analyzed.

**Results:**

All patients underwent biopsies under general anesthesia with an average surgery time of 29.5 ± 4.5 min. The final pathological diagnostic accordant rate with the preoperative ones was 100%, and the pathologic examination of our patients showed features of diffuse large B-cell lymphoma. During the surgery, one patient suffered intratumoral hemorrhage without leading to serious cerebral edema, and conservative treatment was given. There was no death occurring during the study, and there were no significant differences in the Karnofsky Performance Scale Scores of all patients before and after surgery. Finally, they were transferred to the hematology department for standardized chemoradiotherapy according to the pathological results of PCNSL.

**Conclusion:**

This study shows that it may play a vital role in the early diagnosis of PCNSL with the technique of multimodal imaging. The technique of frameless robotic stereotactic biopsy for obtaining the pathology outcomes in suspected PCNSL patients has the advantages of safety, efficiency, and minimally invasiveness.

## 1 Introduction

Primary central nervous system lymphoma (PCNSL) is a rare intracranial malignancy that belongs to highly aggressive extranodal non Hodgkin lymphoma and has a poor prognosis [1, 2]. PCNSL tends to occur in older patients, with an annual incidence of about five parts per ten millions. The lesions often involve the brain, show infiltrative growth, and are often difficult to identify with brain glioma and brain metastases on imaging. However, PCNSL is relatively sensitive to chemoradiotherapy and has a relatively significant treatment effect, so early diagnosis and definite pathology are particularly important [3, 4, 5, 6]. Brain stereotactic biopsy surgery is the gold standard for the diagnosis of PCNSL in the brain [7, 8]. With the development of multimodal imaging technology and continuous update of stereotactic devices [7, 9], combining the two effectively in clinical applications, under the premise of more accurate imaging diagnosis, needle biopsy of PCNSL patients using frameless stereotactic robot is performed to clarify the pathology, and provide treatment basis for subsequent chemoradiotherapy.

## 2 Patients and methods

### 2.1 Subjects and the clinical data

The clinical data of eight patients with suspected intracerebral PCNSL who were diagnosed by multimodal imaging, we retrospectively reviewed They were admitted to the Department of Neurosurgery, Nanjing Hospital, Nanjing Medical University between January 2020 and December 2021. The period of follow-up interviews ranged from 2 to 10 months after discharge, follow-up was conducted by means of telephone calls and outpatient clinic visits. The endpoint of the follow-up study was March, 2022.

### 2.2 Surgical approach

**Figure 1:**
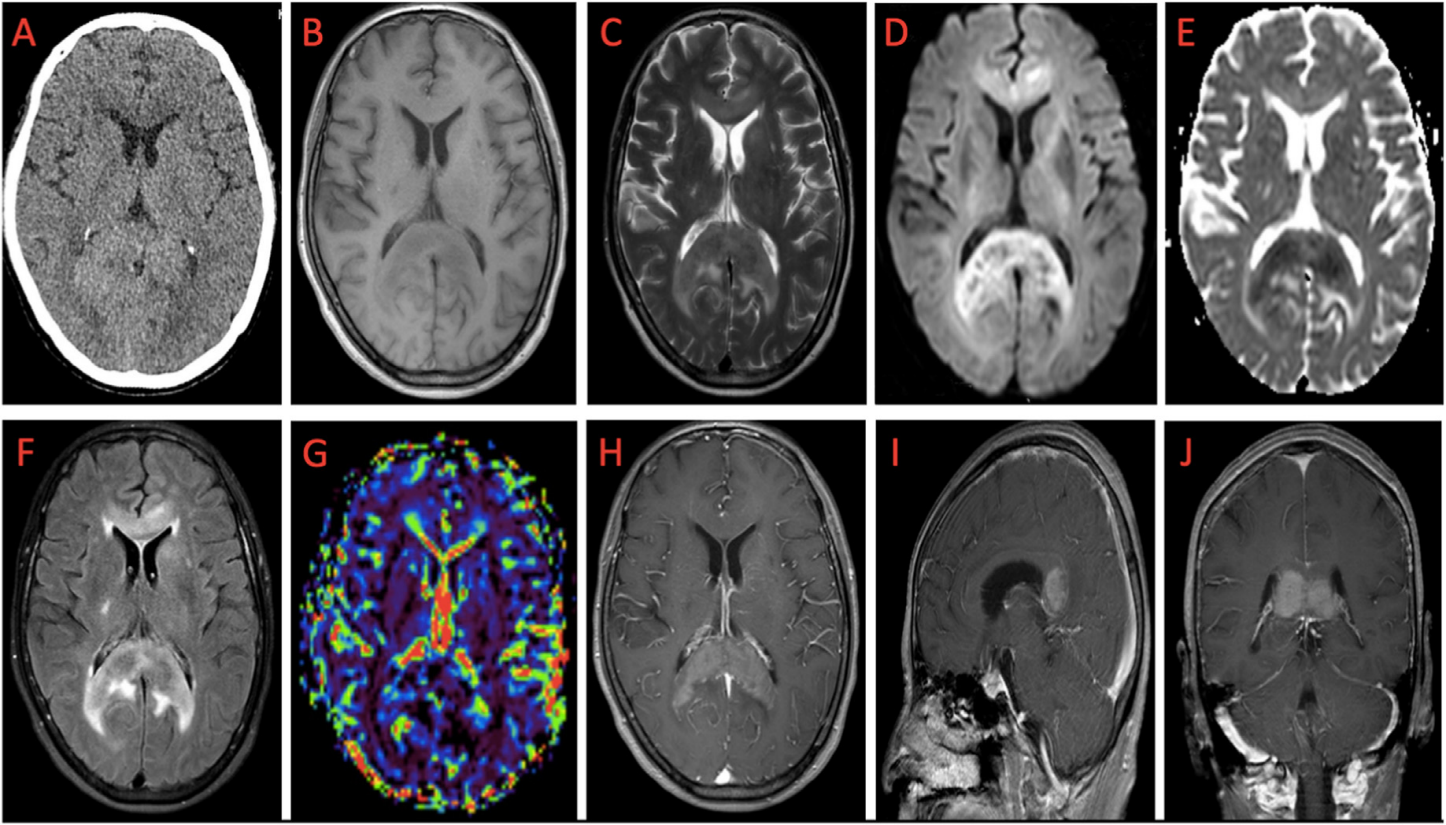
Multimodal imaging findings of a patient with primary CNS lymphoma in the splenium of the corpus callosum: A: slightly higher CT plain scan density; B: hypointense in T1; C: hypointense in T2; D: hyperintense in DWI; E: hypointense in ADC; F: hyperintense in FLAIR; G: hypoperfusion in Perfusion; H–J: Pterygoid, symmetrically distributed, heterogeneous enhancement was seen on axial, sagittal and coronal T1 enhancement.

**Figure 2:**
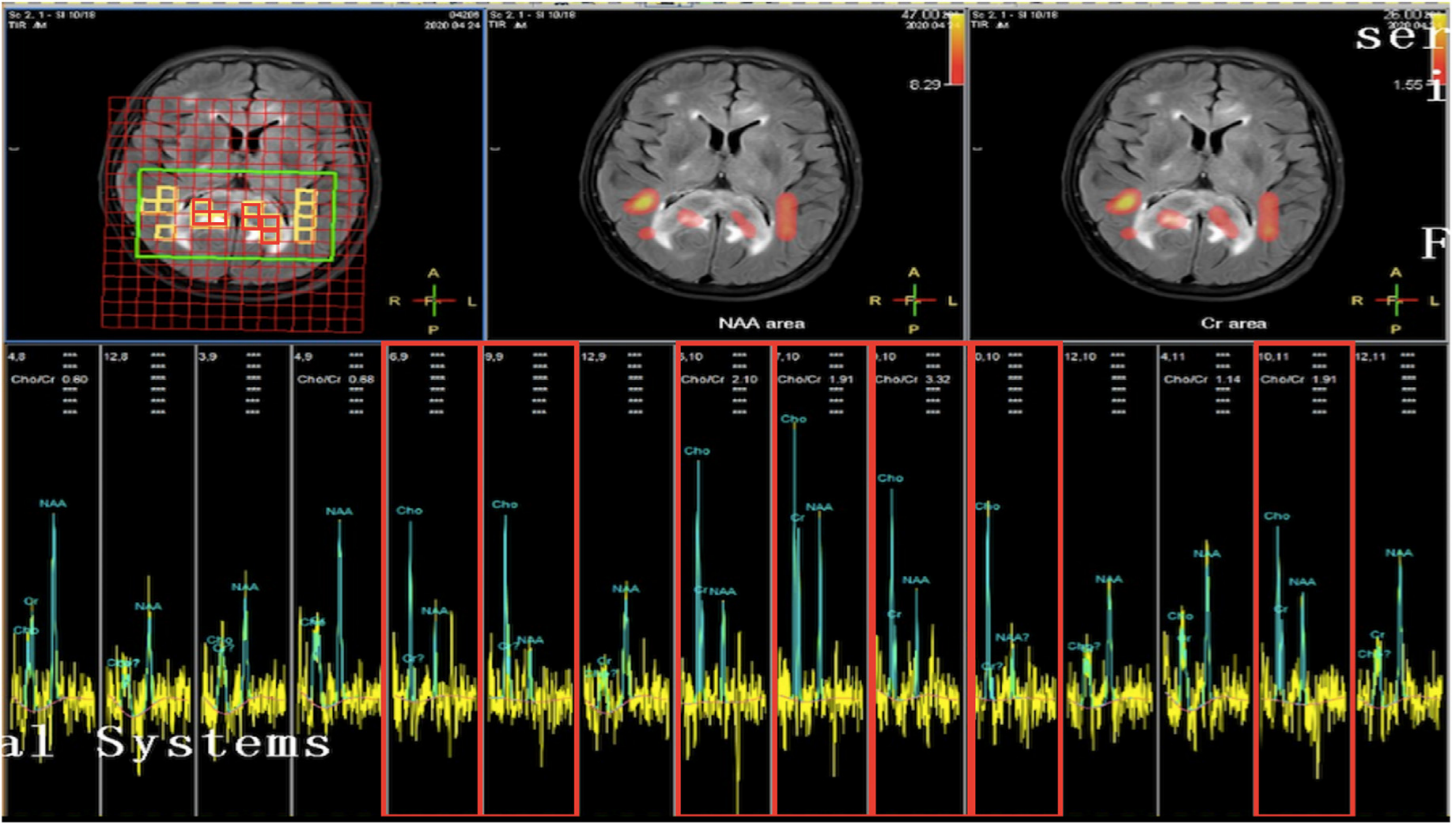
The spectra showed an obvious increase in CHO, a decrease in NAA, and an increase in CHO/Cr and Cho/NAA ratios in the lesion (in the red boxes) compared with the normal brain (in the white boxes).

All patients underwent multimodal imaging, such as: head CT (Figure 1A), unenhanced (Figure 1B–F) and contrast enhanced head MRI scan (Figure 1H–J), MRI perfusion (Figure 1G) and MR-spectroscopy (Figure 2). They were performed as needed to help to distinguish PCNSL from the intracranial tumors, especially gliomas and metastatic tumors. The patients were also given routine pre-operation preparations and informed consent about the surgical approach and surgical risks. They also accepted conventional therapy except hormone therapy. On the operation day, the patients underwent thin slice CT scanning after four spherical visible markers were attached on the scalp. The CT scan used a continuous axial scan, the slice thickness was 0.625 mm, the interval was 0 mm, and the scan time was 1.2 s. The needle biopsy surgery was performed with a frameless stereotactic robot (China-made Cas-R-2 robotic system).

Patients were placed in the corresponding supine or lateral decubitus position according to the place of tumor with the head Fixed using a Mayfield clamp after general anesthesia.

**Figure 3:**
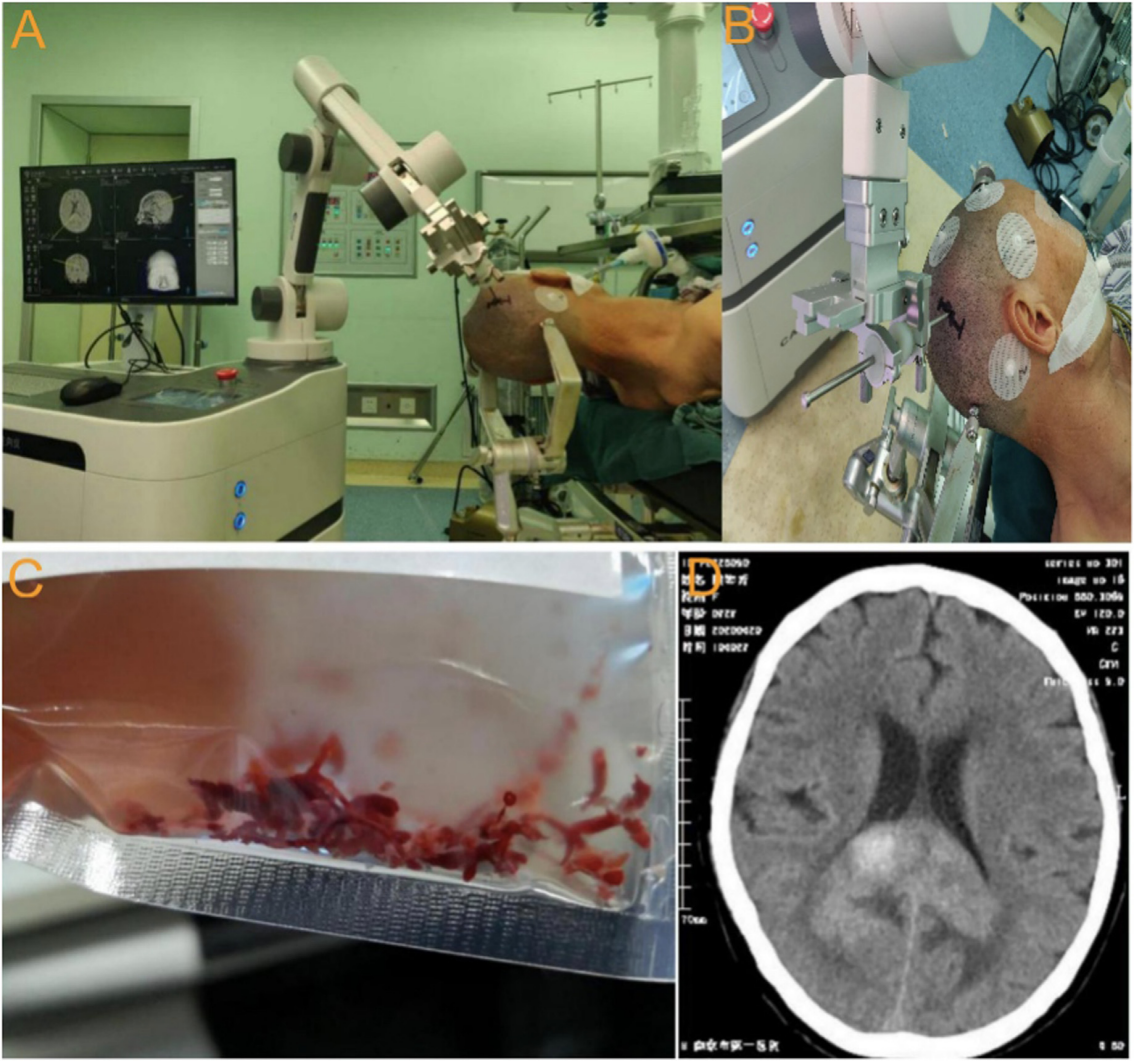
Procedure of frameless stereotactic robotic needle biopsy in the patient with PCNSL: A: the puncture trajectory was determined according to the puncture target; B: Four spherical CT-visible markers in the fusion image were set as landmarks; C:Needle Biopsy specimens were obtained; D: Review of head CT on the first postoperative day showed that there was a small amount of haematoma in the tumor cavity, was caused by the procedure of puncture.

The stereotactic robot consists of a positioning system that has been widely used in clinical practice and offers visual recognition for the needle biopsy surgical operations. The needle-threading stab of the robot can reach the target under the guidance of the positioning system, the accuracy was tested through the following steps: 1). The data of head CT and previous MRI images are imported into the computer software system of the unframed stereotactic robotic device, so the fusion image would show a picture of the three-dimensional image of the patient’s head and the exact location of the lesion could be accurately calculated. Meanwhile, the appropriate positions of the marker and target point are marked on the images. 2). The target point, optimal entry points, and trajectory of the biopsy were designed and planned using the computing system of the robot. The surgical trajectory should be designed to avoid the sulci and enclosed vessels to reduce the incidence of intracranial hemorrhage, meanwhile, it should also avoid the ventricles to reduce the loss of cerebral fluid to prevent the trajectory deviating from the original target. 3). The four spherical CT-visible markers in the fusion image were set as landmarks to register the position in the scalp of patients with preoperative plans (Figure 3A and B).

### 2.3 Surgical procedure

After a precise entry point has been designed, the surgical trajectory is established at each anatomical layer from the scalp to the dura. A burr hole of about 1 cm would be needed for the needle biopsy after the incision of the dura. The dural opening should be made to ensure that the trajectory would avoid the margins of the burr hole. Its size should be small enough to prevent excessive loss of CSF, meanwhile, it should be large enough to admit the stereotactic probe. Then the puncture needle with the lateral hole was placed at the end of the mechanical arm of the robot, it moves according to the surgical planning system with the puncture needle aiming towards the surgical target. Then, the needle was used to puncture the target of the tumor in a standard fashion. The inner sheath was rotated and a little tumor mass was sucked and cut in the needle. Every time, a lesion mass measuring about 10 mm × 1.5 mm × 1.5 mm could be obtained (Figure 3C). After we got enough tumor mass for the need of pathology, the needle was removed and the incision was sutured layer by layer.

Postoperative treatment: All patients received the treatment of dehydration and preventive hemostasis for 3 days.

The head CT was performed on the first and fourth postoperative days to determine whether intratumoral hemorrhage occurred (Figure 3D).

## 3 Ethics statement

This study was conducted in accordance with the declaration of Helsinki for Medical Research involving Human Subjects- Ethical Principles for Medical Research Involving Human Subjects and it was conducted with approval from the Ethics Committee of Nanjing First Hospital. Written informed consents were obtained from all patients.

## 4 Result

### 4.1 Clinical data of PCNSL patients

All patients underwent core needle biopsy surgery with stereotactic robot under general anesthesia. There were 5 males and 3 females, aged 52–75 years, with an average of 65.0 ± 8.7 years. The main clinical symptoms were dizziness, headache, limb weakness, unresponsiveness and vomiting. The median duration of the clinical symptoms ranged from 4 days to 20 days (see Table 1).

**Table 1:** Clinical data of PCNSL patients.

Patient	Sex	Age (years)	Lesion locations	Clinical symptoms	Course of disease (days)	KPS Scores before operation	Surgery time (minutes)	KPS Scores after operation
1	male	72	paraventricular, right thalamus, tubera corporis callosi, genu of corpus callosum	limb weakness, slow response	7	70	31	70
2	female	72	tubera corporis callosi	dizziness and vomiting	14	60	37	60
3	male	52	left parietal occipital lobe	limb weakness	20	80	28	80
4	male	61	right basal ganglia, brain stem	fatigue, blurred vision	14	60	33	60
5	male	70	left parietal occipital lobe	right lower limb weakness	10	80	24	80
6	male	75	right frontotemporal lobe and insula	headache	7	70	32	70
7	female	64	right frontal lobe	headache	14	90	26	90
8	female	54	right temporal lobe	headache and vomiting	4	80	25	80

KPS:Karnofsky Performance Scale.

### 4.2 Multimodal imaging findings of PCNSL patients

PCNSL is isodense on plain CT scan (Figure 1A). Generally, MRI was an important imaging modality in the diagnosis of patients with GBM or PCSNL, including contrast-enhanced T1-weighted sequences (WI). PCNSL always showed medium or slightly low signal in T1-WI (Figure 1B) and T2-WI (Figure 1C) with fist-like, lumpy or nodular enhancement. There is always strong homogenous enhancement (Figure 1H–J) after contrast injection and low MRI perfusion (Figure 1G) in PCNSL patients (see Table 2).

**Table 2:** Multimodal imaging findings of PCNSL patients.

Multimodal imaging	signal intensity
CT plain scan density	isodense
T1	hypointense
T2	hypointense
DWI	hyperintense
ADC	hypointense
Flair	hyperintense
Perfusion	hypoperfusion
MRS	Cho↑, Naa↓
CHO/Cr ratios	high
Cho/NAA ratios	high

### 4.3 The result of complications

The surgical procedures were performed on 8 patients, of which 7 were successful. The average time of the operation was 29.5 min, which ranged from 24 to 37 min. There were no significant differences in the Karnofsky Performance Scale (KPS) Scores of all patients before and after surgery. One female patient developed a small amount of haematoma in the tumor cavity on the first postoperative day, which was caused by the procedure of puncture. The patient had no obvious new onset or aggravation of symptoms, and she was treated with conservative observation. The remaining 7 patients had no de novo intracranial hemorrhage on postoperative routine CT examination. None of the patients experienced postoperative infection or death.

### 4.4 The result of pathology

A total of 8 patients were diagnosed as suspected PCNSL by the multimodal imaging preoperative, all specimens containing a highly cellular infiltrate of malignant cells and the pathological results were diffuse large B-cell lymphoma postoperative. All of the cases were consistent with the result of pathology, so the coincidence rate was 100% (8/8).

### 4.5 The result of the follow-up study

All patients were transferred to the hematology department for further treatment when the results of pathology come out. The overall follow-up rate was 100% (8/8), and all patients were alive at the end of the follow-up period.

## 5 Discussion

PCNSL accounts for about 5% of all brain tumors and mostly typically presents as a solitary mass. It is always located in the most common regions of involvement including the cerebral hemispheres, basal ganglia, thalamus and corpus callosum [1, 10, 11]. It is important to know the imaging features of PCNSL with multimodal imaging findings. On MRI perfusion imaging, PCNSL is always lower than other intracranial tumors, such as meningiomas, metastases and high grade gliomas. It is due to the low induction of tumor neovascularization and increases in vascular permeability after the destruction of the blood–brain barrier [12, 13, 14]. Therefore, the edge of the enhancement in the tumor is often relatively blurred and the surrounding edema is obvious. Meanwhile, on the DWI image, the lesion shows hyperintensity suggesting restricted diffusion. Because the tumor cells in PCNSL are always dense with less cytoplasm and a high cytoplasmic ratio, there was small extracellular space in them. The diffusion of water molecules is always limited in tumors [15, 16]. Furthermore, Because the blood supply in PCNSL is not rich, it represents low perfusion with decreased cerebral blood volume and cerebral blood flow, and the average passage time and peak time would be prolonged on PWI image [17]. The image in PCNSL was significantly different from the high perfusion of high-grade gliomas. It was also reported that the CBV values without contrast-deficient correction would be one of the best indicators for identifying PCNSL and gliomas by PWI [18, 19].

In the brain magnetic resonance spectrum (MRS), the reduced NAA peak generally reflects neuronal loss or dysfunction and the elevated Cho peak usually represents that the cell membrane is renewed quickly and the cell density is high. The hallmark of MRS findings of PCNSL is the typical image just like other malignant intracranial tumors: Cho peak is significantly high, NAA peak is significantly low and the Cho/NAA ratio is high [20, 21].

PCNSL is a high-mortality disease characterized by high invasiveness and rapid progression. So, early detection, early diagnosis and early treatment are crucial for patients with PCNSL. The accurate determination of pathology related to diagnosis is of great help for the best therapeutic schedule, which can guide treatment and improve the survival status of these patients. At present, it is generally believed that PCNSL is sensitive to chemotherapy and radiotherapy. High-dose methotrexate-based chemotherapy is the first-line treatment for the majority of the diagnosed PCNSL patients, and some patients would receive chemotherapy-based comprehensive treatment or adjuvant radiotherapy, as appropriate [22, 23, 24, 25]. In the study by Caroline Houillier, et al., the increasing proportion of elderly patients within PCNSLwas found to be associated with poor outcomes. Patients aged over 60 years accounted for 72% and patients aged over 70 years accounted for 43% [26]. In our study, patients aged over 60 years accounted for 75%, including 50% of patients aged over 70 years. They raised the need for a comprehensive pretreatment clinical evaluation and therapeutic management. In contrast, there is a good therapeutic effect and a higher rate of long-term survival in younger PCNSL patients, who can benefit from high-dose chemotherapy with autologous stem cell transplantation [26]. Most PCNSL patients can be preliminarily diagnosed by multimodal imaging technology, but it is still difficult to completely rely on the techniques to make a final diagnosis. Definite diagnosis of PCNSL always relies on pathological examination. Frame-based, stereotactic craniotomy localization would be an accurate method to get the biopsy pathology even molecular pathology [27]. It can not only provide a reliable basis for targeted therapy and immunotherapy, but also it has the low rate of postoperative neurological deficits and complications [28, 29, 30]. The robotic system was first used in neurosurgery in 1985 [31]. With the continuous development of stereotactic and neuronavigation technologies, robot-assisted frameless stereotactic systems are reconsidered to have numerous advantages, such as accuracy, accurate spatial positioning, high neurosurgical precision, consistency, never fatigue, while compared with the manual approach [32, 33].

Meanwhile, there are a lot of systematic reviews and meta-analyses about comparisons of frame-based versus frameless in intracranial stereotactic biopsy. It was reported that the frameless biopsy technique was as efficient, accurate and safe as the frame-based brain biopsy technique with a low complication rate. There were no significant differences existed in diagnostic yield, morbidity, and mortality between them. However, frameless biopsy can alleviate the suffering of the patient and reduce the total duration of the procedure. Remebot robot-assisted frameless brain biopsy is easy to use and better accepted by patients than frame-based biopsy. Frameless biopsy is associated with shorter procedural times relative to frame-based biopsy, and it may have wider indications in brain biopsy due to its high flexibility [34, 35].

The successful biopsy always has advantages as below: more precise localization, less chance of bleeding, less terrifying anxiety, less time-consuming. More specifically, it could be defined as the following:

1. The adequate tissue of the intended target was sufficient and typical, and a diagnosis could be established for the reporting pathologist.
2. The successful control of biopsy-induced bleeding was achieved during the procedure in all patients.
3. The procedure could be as comfortable as possible to alleviate the suffering of patients.
4. It could greatly simplify the operation procedure, reduce surgical time, and minimize the rates of intracranial infection with less exposure time.

It was reported that 190 infratentorial stereotactic biopsies of posterior fossa tumors were performed with a stereotactic frame under local anesthesia in a semi-sitting position by Jacek Furtak. 4% of patients had minor complications and 1.5% had major complications in their study. One patient died from intracranial bleeding. Nine biopsies were non-diagnostic [36]. In our study, the patients were placed in the corresponding supine or lateral decubitus position according to the place of the tumor with the head fixed using a Mayfield clamp. They underwent surgery with general anesthesia instead of local anesthesia. We wished it would reduce the pain and psychological burden, and the procedure could be as comfortable as possible to alleviate the suffering of the patient. Compared with the conventional frame stereotactic technique, the surgical planning system of neurosurgery frameless stereotactic robot can represent three-dimensional volumetric reconstruction of the lesion and important surrounding neurovascular structures to design a safe and accurate puncture path. On the one hand, it can overcome the limitations of the traditional frame and reduces the pain and psychological burden of patients with a frame on their heads. On the other hand, it can increase the safety and flexibility of surgery. The frameless stereotactic robot does not need any additional instrument which is used by the neuronavigation system. After the surgical target and puncture path were planned and established, the robotic arm could perform the correct positioning puncture immediately. Preoperative preparation would be simpler and intraoperative manipulation would be easier. It greatly simplifies the operation procedure, reduces the surgical time, and minimizes the chance of bleeding caused by the puncture and the rates of intracranial infection with less exposure time [9, 37, 38, 39]. In this study, the average surgical time was 29.5 min, and there were no cases of intracranial infection postoperatively.

There are some notices during the surgery with the neurosurgical frameless stereotactic robot. First, during the preoperative CT examination, the attached four spherical visible markers on the head should surround the lesion. The four markers should not be arranged in a line array and not in the same plane, and the nearby scalp should be kept flat in case of the fixed marker shifting. Second, it would be better to choose the central area of the lesion as the puncture target instead of the areas of necrosis or calcification, and it is necessary to avoid the important structures such as the sulcal vessels, ventricles, cisterns, and functional areas when designing a biopsy puncture path strategy [29]. Third, it was vital to learn whether there was hematocele or active bleeding inside the lesion at the end of surgery by observing the puncture needle [40, 41]. After confirming that there was no bleeding, the needle could be withdrawn slowly. In case of bleeding occurred intraoperatively, the outer cannula of the puncture needle should be kept for the drainage of blood out of the brain and 500–1000 U of thrombin (dissolved in 2–5 mL of saline) could be injected into the target lesion to promote coagulation [42, 43]. Immediate follow-up CT was performed to check the area of the hematoma to decide the further treatment plan.

Neurosurgery frameless stereotactic robots have been widely used in various types of neurosurgery, some aspects still need to be improved. One of the most common complications of needle biopsy is intracranial hemorrhage resulting from direct needle injury to a vessel [41]. It is necessary to get enough specimens to perform the pathological examination and molecular analysis, which is bound to increase the number of punctures and the risk of bleeding. Conversely, if only a small number of pathological specimens are obtained, they would be insufficient for pathological diagnosis. Even more, sometimes the specimens are mixed with necrotic tissue or normal brain tissue, which would increase the difficulty of the pathological diagnosis [44, 45]. In summary, the imaging findings of PCNSL are complex, but multimodal imaging techniques can improve the accuracy rate of preoperative imaging diagnosis. The stereotactic robotic needle biopsy surgery has many advantages, such as simple manipulation, less surgical trauma, short time-consuming, precise and safe.

The study about multimodal imaging combined with frameless robotic stereotactic biopsy in the diagnosis of PCNSL fully reflected a precise, efficient, minimally invasive safe modern neurosurgical concept, with better clinical application. PCNSL is a disease with a low incidence rate. Limitations of the study include the relatively small cases (only 8 cases) and short term observation. Further large, prospective, randomized studies are needed.

## Declarations

### Author contribution statement

Yong Tang; Yan Shi: Performed the experiments; Wrote the paper.

Ling Wang: Performed the experiments; Analyzed and interpreted the data; Wrote the paper.

Zheng-ting Qian: Analyzed and interpreted the data.

You-wu Fan: Contributed reagents, materials, analysis tools or data.

He-ming Wu; Xiang Li: Conceived and designed the experiments.

### Funding statement

Dr. He-ming Wu was supported by National Natural Science Foundation of China [No. 81301180], Jiangsu Commission of Health [M2020046].

### Data availability statement

Data included in article/supp. material/referenced in article.

### Declaration of interest’s statement

The authors declare no competing interests.

### Additional information

No additional information is available for this paper.
